# Interpersonal attunement in social interactions: from *collective* psychophysiology to *inter-personalized* psychiatry and beyond

**DOI:** 10.1098/rstb.2021.0365

**Published:** 2023-02-13

**Authors:** Dimitris Bolis, Guillaume Dumas, Leonhard Schilbach

**Affiliations:** ^1^ Independent Max Planck Research Group for Social Neuroscience, Max Planck Institute of Psychiatry, Kraepelinstrasse 2–10, Muenchen-Schwabing 80804, Germany; ^2^ Centre for Philosophy of Science, University of Lisbon, Campo Grande, 1749-016 Lisbon, Portugal; ^3^ Department of System Neuroscience, National Institute for Physiological Sciences (NIPS), Okazaki 444-0867, Japan; ^4^ Precision Psychiatry and Social Physiology Laboratory, CHU Ste-Justine Research Center, Department of Psychiatry, University of Montreal, Quebec, Canada H3T 1J4; ^5^ Mila - Quebec AI Institute, University of Montreal, Quebec, Canada H2S 3H1; ^6^ Culture Mind and Brain Program, Department of Psychiatry, McGill University, Montreal, Quebec, Canada H3A 1A1; ^7^ Department of Psychiatry and Psychotherapy, University Hospital, Ludwig Maximilians Universität, Munich 40629, Germany; ^8^ Department of General Psychiatry 2, LVR-Klinikum Düsseldorf, Düsseldorf 80336, Germany

**Keywords:** social interaction, self, culture, interpersonal attunement, collective psychophysiology, inter-personalized psychiatry

## Abstract

In this article, we analyse social interactions, drawing on diverse points of views, ranging from dialectics, second-person neuroscience and enactivism to dynamical systems, active inference and machine learning. To this end, we define interpersonal attunement as a set of multi-scale processes of building up and materializing social expectations—put simply, anticipating and interacting with others and ourselves. While cultivating and negotiating common ground, via communication and culture-building activities, are indispensable for the survival of the individual, the relevant multi-scale mechanisms have been largely considered in isolation. Here, *collective psychophysiology*, we argue, can lend itself to the fine-tuned analysis of social interactions, without neglecting the individual. On the other hand, an interpersonal mismatch of expectations can lead to a breakdown of communication and social isolation known to negatively affect mental health. In this regard, we review psychopathology in terms of interpersonal misattunement, conceptualizing psychiatric disorders as disorders of social interaction, to describe how individual mental health is inextricably linked to social interaction. By doing so, we foresee avenues for an *inter-personalized* psychiatry, which moves from a static spectrum of disorders to a dynamic relational space, focusing on how the multi-faceted processes of social interaction can help to promote mental health.

This article is part of the theme issue ‘Concepts in interaction: social engagement and inner experiences’.

The true direction of the development of thinking is not from the individual to the social, but from the social to the individual.

Lev Vygotsky

## 1. Interpersonal attunement in and through social interaction

People tend to ‘fall in synchrony’ in social interactions, e.g. through spontaneously aligning rhythmic behaviours, such as gait and clapping or adopting others' mannerisms, from shaking a foot or scratching the head to adopting each other's speech styles, emotions and moods [1–6]. Intriguingly, humans oftentimes attune to each other beyond personal cost-benefit evaluation (e.g. informal social norm following; [7]) and even against their actual intention [8]. Furthermore, interpersonal attunement in social interactions has not only been demonstrated at the behavioural, but also at the neural level, including phenomena ranging from motor synchrony [9] and shared psychological perspectives [10], to collective musical experience [11] and real-life social relationships [12,13].

All of these instances of interpersonal attunement can be thought of as facilitating human communication, collaboration and eventually trust-based social relationships, by virtue of locally decreasing interpersonal complexity [7,14], while providing new tools of self-regulation at the individual level. Indeed, from developmental psychology to social neuroscience and psychiatry, an increasing number of findings have demonstrated the critical role of interpersonal coordination in the development and maintenance of our social—and even individual—abilities. It is through co-ownership of social interactions that babies learn the distinction between self and other [15], the temporality of social exchanges [16], and the notion of a shared world [17]. Later on, it is through engagement in social interaction that cognitive abilities such as language and ‘theory of mind’ emerge, leading to the ability to effectively communicate and make sense of one's own and other persons' intentions and beliefs, but also to the generation of certain social biases associated with expectations towards social norms [18,19]. In a nutshell, interpersonal attunement in early social interactions lay the ground for human becoming, via the co-construction of progressively symbolic communication and its internalization as inner speech [20,21].

In this regard, our view is inspired by and consistent with descriptions provided by Vygotsky, who already at the beginning of the previous century suggested that all ‘higher’ mental processes *within* an individual result from an internalization of prior social interactions *between* people [21]. Furthermore, he proposed that every mental function of this kind appears twice in a child's development, first at a social level (i.e. ‘intermind’) and then at an individual level (i.e. ‘intramind’): in brief, he suggested that ‘through others we become ourselves’. Taken together, interpersonal attunement, here, is defined as the set of the multi-scale processes of establishing joint and eventually collective interaction patterns and expectations about states of the world and social behaviour [19,20]. Such interpersonal attunement, in turn, enables and facilitates intrapersonal attunement and vice versa, here, taken as the set of self-regulation processes (e.g. inner speech and interoception [21,–23]). Critically, interpersonal attunement does not only include harmonic developments, but also at times dramatic tensions and conflicts that appear instrumental in driving change across the entire lifespan [20]. In this light and leaning on diverse perspectives, ranging from second-person neuroscience, dialectics and enactivism to dynamical systems, active inference and machine learning, in this article, we argue that a multi-scale, fine-grained analysis of social interaction might help us to elucidate the underlying behavioural and neural mechanisms both at the level of the individual, but also at the level of interacting bodies and minds.

More concretely, in the current section, we discuss the role of *interpersonal attunement* in social interactions and how it shapes the formation of the self. In the second section, we describe how psychopathology can be construed as *interperpersonal misattunement*. In the third section, we describe the paradigm of *collective psychophysiology* as a methodology to empirically study interpersonal (mis)attunement. In the fourth section, we suggest an integrative clinical, empirical and computational research line, which aims at formally defining a multi-dimensional relational *space of conditions*, embracing different levels of analysis and how developments could contribute to what could be described as an *inter-personalized psychiatry*. We end this article by describing some of the societal implications of our approach.

### (a) The dialectics of internalization and externalization

In this first section, we place our focus on the individual, reviewing human development and becoming as the dynamic interplay between (social) internalization and (collective) externalization in and through social interaction [20–25]. On the one hand, internalization, here, can be thought of as the co-construction of bodily structures actively reflecting the social world and the organism. On the other hand, externalization is taken as the collective construction of the world, including other (inter-)bodily structures. Along those lines, the collective and the level of the individual appear inextricably linked, as an interrelation between the formation of multi-scale hierarchical bodily structures (e.g. psychophysiological states) and interpersonal statistical regularities beyond the individual (e.g. social norms). Below we further unpack the key notions of internalization and externalization, while connecting them to recent computational theories of brain and bodily function.

Our notion of internalization is based on the Vygotskian take, thought of as the internal reconstruction of an external operation [21], further operationalized within the predictive processing framework ([26]; see also [27]). Predictive processing has been defined as a hierarchical bidirectional process through which an organism adjusts itself in order to (Bayes) ‘optimally’ predict environmental and bodily regularities. With regard to brain function, predictions are continuously generated and propagated from higher levels of the neural hierarchy to lower ones in an attempt to explain away so-called prediction errors, i.e. the discrepancy between incoming information and generated predictions. On the other hand, prediction errors are propagated from lower levels of the hierarchy to higher ones in order to ‘optimally’ reconfigure the organism. Of note, in the framework of predictive processing, higher (deeper) levels of the neural hierarchy are thought of corresponding to higher levels of abstraction. In brief, organisms are continuously trying to optimize their expectations, via minimizing overall prediction error, across various scales, through functions such as perception and learning. In doing so, organisms maximize their odds of survival via prediction error minimization. An organism is assumed to achieve this by virtue of keeping—at least temporarily and locally—entropy low, or, in other words, the states the organism can visit as a system bounded (e.g. body temperature around 37°C). Notably, expectations in this framework cover a wide spectrum of controlled processes, from conscious actions and thoughts to ‘automatically’ adjusted interoceptive states.

Yet, humans are not mere passive spectators who just assimilate reality. On the contrary, they actively interact with their world, including other persons, modifying it to meet their expectations through processes of externalization (cf. active inference; [22,28]). For instance, when the perceived partial pressure of carbon dioxide surpasses certain bounds, the respiratory system is in charge of keeping it within expected levels, preserving bodily order and thereby survival. To give another example, when a person sits in the cold for an extended period of time, their body temperature tends to fall below predicted values. Trembling, lighting a fire, or entering a warm facility often reverses this trend and aids in keeping the body temperature within boundaries that are conducive to survival and well-being. Such prediction error minimization processes of actively controlling the body and the environment, with the goal of actively transforming the world and the body such that they conform with prior expectations, has been referred as active inference ([26,28]; see also [27]).

Importantly, in real life, the above-mentioned processes of internalization and externalization should be considered as inextricably linked. To this end, in the next section, we will situate these accounts in the sociocultural realm through a dialectical prism, emphasizing what we describe as ‘becoming-with’ (on a collective level) and its interrelation with ‘being’ (on an individual level).

### (b) The dialectics of the individual and the collective

The above-mentioned hierarchical structures of predictive processing, in our view, should be considered as collectively shaped. First, we dynamically ‘embody’ each other in and through social interaction [20], enabling interpersonal attunement (e.g. interpersonal belief resonance; [29]). In other words, by engaging in sensory-motor couplings with others in social interactions, we have our bodily structures mutually transformed beyond the here and now [20,30]. Second, such structures, arguably, unfold within nested time and space scales, from biology and cognition all the way up to society [14,31,32]. That is, these multi-scale dynamics encompass bottom-up and top-down processes, even outside the skull of individuals, and thus solving such dialectic requires accepting the complementary nature of reduction and emergence [33]. Various theoretical frameworks have been proposed, from autopoïesis and enaction [34] to coordination dynamics [35] and active inference [26].

While autopoïesis is one of the first examples of such multi-scale frameworks, its attempt to connect biology up to the social level has been limited. Indeed, first-order structural coupling allows for the emergence of cells, and second-order structural coupling allows for the emergence of multi-cellular organisms; social interaction between those complex organisms, in turn, can be thought of as a third-order structural coupling [36]. However, the (bio)logical grounding of autopoïesis assumed a strong solipsism regarding knowledge, and thus the largest autopoïetic unit has always remained a single agent [37,38]. In fact, this constitutes a key point of disagreement between Maturana and Varela, as well as the later developed paradigm of ‘enaction’, which on the one hand, keeps the inspiration from autopoïesis and on the other hand, aims to explore the link between our individual experience and the interaction—and even co-existence—with others [39].

The coordination dynamics framework had relatively less problems for bridging the micro–macro divide, since it comes from the language of complex systems and nonlinear dynamics. Originally applying the tools of synergetics to the understanding of finger movement [40], the derived principles were revealed to apply across multiple scales: between brain regions within a given brain, between limbs within a given individual and even between individuals within social groups [33].

The frameworks of predictive processing and active inference have lent themselves to the formal generation and comparison of hypotheses through their associated generative models [41–44], yet also have been criticized for an overly passive and detached approach to make sense of human cognition in certain articulations [45]. Additionally, more broadly the field of computational psychiatry [46] can be questioned for largely emphasizing certain aspects of (artificial) cognition (e.g. reinforcement learning, decision-making, as well as representations of reward, punishment and risk as optimization functions), at the expense of other ones (e.g. social dimension, subjective experience and creativity). Therefore, while active inference appears as a potentially unifying framework, a lot of work remains to be done toward a balanced state space which would account for a realistic perspective of the human mind. Having said that, despite their original limitations, within the relevant scientific landscape, the active inference frameworks can be viewed as a powerful toolbox, which shares some neurobiological grounding with autopoïesis and enaction, while at the same time deploys a physics formalism akin to coordination dynamics [26,47].

Considering the commonalities (the emphasis on complex systems, multi-scale dynamics, uncertainty, embodiment and self-organization to name but a few; [20,25,45,47–55]), but also the tensions between the various flavours [48,52,56,57], we foresee a dynamic convergence between the above-mentioned frameworks, which, in our view, have been already capturing complementary—overlapping at times—projections of the multi-scale dynamics of social interaction and the mind. As a result of their interaction, on the one hand, enaction has been drawing inspiration from the commitment to formalism [58], while, on the other hand, active inference has been increasingly situated within multi-scale (social) interactions [20,59–62].

In doing so, active inference has been aspiring to become a generalized framework in various fields, ranging from philosophy, psychology and psychiatry to neuroscience, robotics and artificial intelligence (e.g. [23,52,63–67]; ‘a theory of every “thing” that can be distinguished from other “things” in a statistical sense’ as Friston provocatively puts it [68]; but also note critiques on the scope of current versions of the framework; [69–71]).

In light of the above-described considerations, active inference, we argue, should not be thought of as exclusively lying within or being restricted to the individual. For example, social norms, architecture and technology may all be understood as a collective effort to optimize predictability via transforming ourselves and the environment in accordance with bodily and interpersonal expectations [20,72,73]. Here, bodily expectations could be an adaptive range of certain attributes of a living human body, such as temperature and pressure, but also psychological states, such as the desire for socializing and reproducing, while interpersonal expectations may include whatever another person or a group of persons could expect from us (cf. social norms). We typically try to satisfy both bodily and interpersonal expectations, even if they, at times, appear contradictory (cf. the various conflicts between desires and cultural conventions). Importantly, both types of expectations should be considered as dynamic states of the world and thus ‘historical’ products of interaction. In other words, expectations are multi-scale processes, fluctuating at various scales, from phylogeny and ontogeny to culture and individual psychophysiology.

Here, let us examine an illustrative hypothetical scenario on the ontogenic scale, inspired by Vygotsky [21]. A baby, trying to regulate her hunger and thus interoception balance, tries to reach for food. However, she is unable to do so, despite stretching her whole body, even extending the index finger. A caregiver, observing the situation and predicting the baby's goal, brings the piece of food closer to the kid. After multiple repetitions, this statistical regularity of interpersonal coordination can be internalized by the baby, who at some point understands that an extended index finger towards an object denotes the intention of directing the attention of another person to an object in the environment. This is an important realization, as it is in this very process of transforming the interpersonal into an individual mental process, that the baby masters a completely new (mental) tool, through which she can then affect the environment, including others, and critically her own self (cf. self-regulation), in a much more efficient way. Such skills at the interface of the collective and the individual may give rise to core aspects of the human self, such as ‘social agency’, which has been thought of as the ‘sense of self that is gained through the perceived control one exerts over the social world’ [74] . As Vygotsky put it ‘the relation between higher psychological functions was at one time a physical relation between people’ [75] ).

Now stretching this example at an intergenerational scale, one could trace back the interpersonal history of various cultural conventions and social norms, which now appear as psychological laws, such as the need to wear clothes even when the weather conditions do not require to do so [20]. Finally, considering possible interactions of these processes at the scale of phylogeny might even allow us to examine the potential social origins and interrelation of certain aspects of human anatomy and human-specific skills. A typical example can be found at the potential multi-scale interplays between the human eye morphology (e.g. ratio of exposed sclera in the eye outline, which allow the other to recognize easier the gaze's direction), the enhanced cognitive abilities for gaze-based interaction and later abstract cognitive skills [20,76]. These sorts of multi-scale and inextricably linked processes of human becoming while attuning to one another and the environment, is what has been described as dialectical attunement [20,22].

Taken together, humans actively co-construct and co-regulate—in interaction with other organisms—their ecosocial niches, so that they increase survival chances of not just the individual, but also the social group and the species as a whole.

## 2. Interpersonal misattunement in and through social interaction

So far, we have considered the importance of interpersonal attunement in social interactions and the formation of the human self. Subsequently, placing our focus on psychopathology, we extend our discussion from attunement to potential misattunement, discussing the *dialectical misattunement hypothesis* [63]. According to this hypothesis, psychopathology can be viewed not as mere (mis-)function within single brains, but also as a dynamic interpersonal mismatch (for a comprehensive review of the phenomenon as well as the relevant psychophysiological processes see [63]). As we will examine below, the primary aim of such an approach is to move beyond the individual in the study of psychopathology, yet without neglecting the tightly connected psychophysiological processes at play.

More concretely, here, misattunement across persons is thought of as a series of disturbances of the dynamic and reciprocal unfolding of an interaction [59]. Such misattunement results in potentially increasingly divergent prediction and interaction styles and vice versa. Prediction and interaction styles are defined as a set of prior expectations and reaction patterns a person dynamically develops in interaction with the world and others, as discussed above (cf. predictive processing and active inference). The above-mentioned misattunement is not only mediated, but at times reinforced by a selectively designed cultural and technological environment, which is typically meant to conform with and satisfy dominant social standards.

A dynamic interpersonal misattunement is—similarly to attunement—expected to unfold across various scales, from seconds to multiple years. First, consider the case of a conversation between two persons. Someone may voice an unexpected opinion or act in an unforeseen manner. In turn, the second person might react defensively. This slight initial misattunement can potentially spiral out of control, while additional factors, such as emotional engagement and internalized social norms might further reinforce such a vicious cycle. An example of such an interacting dyad may consist of an autistic child who, when stressed, might tend to react with repetitive movements and a neurotypical one who has—via exclusively interacting with neurotypical peers—formed a rather strictly tuned set of expectations (cf. ‘narrow priors’ in Bayesian formulation) of what a typical conversational reaction might be.

Furthermore, imagine a human relationship. Short-scale interpersonal disturbances might lead to a cumulative misattunement, which can oftentimes go beyond the conscious will of the interacting parts. In other words, day-to-day misunderstandings—if not timely resolved—can potentially lead to a cascade of interpersonal obstacles and increasing personal dissatisfaction, up to an eventual dissolution of a relationship [77].

Now consider a child who along her whole development repeatedly experiences such interpersonal misattunement. In such a case, a persistent social exclusion might actually exert a higher impact on the development of this person, than an initial atypicality in the generation of expectations and reactions. This is likely to prevent her from naturally developing the knowledge and skills a typical person develops in and through the daily social interactions within a given culture.

Of note, an interpersonal misattunement, as defined above, lies in the *interaction* between the two parties and as such it constitutes a collective phenomenon non-reducible to either of them. Here, it should become clear that our view goes beyond an apparently persistent misconception that the responsibility of such a misattunement *a priori* lies in the individual who might be considered as the atypical one.

With regard to larger groups of persons, this kind of misattunement could even take on a cultural and as such intergenerational form. For example, culturally cultivated beliefs in a given society about a specific group of people might highly impact the effectiveness of interaction between in- and out-group persons, eventually provoking intergroup conflict and vice versa. From a Bayesian perspective, one can imagine social stigma and stereotypes as a strict set of prior beliefs operating on a relatively long timescale. Although certain rigid prior beliefs can be adaptive, potentially serving as useful heuristics for quick and effective decisions, they can turn out to be detrimental to human communication and well-being in the long run by segregating social groups and perpetuating inequalities.

Here, it is important to underline that, in our view, this kind of interpersonal misattunement should be treated as a phenomenon at the intersection of the individual and the collective. Over-focusing on either of the sides can be counterproductive when it comes to grasping complex phenomena. For instance, an initial (medical) condition can lead to a cascade of other ‘comorbid’ conditions, such as depression and anxiety, not through an actual biologically causal link, but through the interplay of an actual condition and social expectations in a given sociocultural context. Let us consider an illustrative example: a person, who, diagnosed as HIV positive, develops depression the years following their diagnosis. In this case, the psychiatric condition of depression might potentially have to be examined more in relation to an interpersonally aversive environment due to social stigma, than the actual medical condition. In other words, taking social interactions seriously helps to re-emphasize the importance of a genuinely biopsychosocial model of health and disease [78] and argues for a systems approach to medicine with a particular emphasis on dyadic social interactions, which form a crucial interface in connecting different factors and levels of descriptions.

So far, we have examined scenarios of misattunement between persons. However, such interpersonal segregations and social interaction disturbances might even take the form of an environmental misattunement for certain groups of people. Let us, here, contemplate a hypothetical scenario: in a world where human height typically exceeds 3 metres, a person of an average height in our world would face severe difficulties in everyday life; even an activity as simple as sitting on a chair in a restaurant would turn into a challenging endeavour. Now contrast this scenario with the everyday life of an autistic person in a neurotypically designed cultural and technological world. Arguably, at least part of their anxiety could be alleviated by reconsidering the design of our living spaces, both real and virtual ones [79–81]. This kind of inextricably linked and multi-scale processes of dynamically disturbed interaction between persons—mediated by the (cultural) environment—is what we call dialectical misattunement [22].

Importantly, the dialectical misattunement hypothesis makes concrete suggestions with regard to social interactions and interpersonal relationships in real life, amenable to empirical validation: in certain scales ‘interactions within homogeneous dyads are expected to appear smoother compared to heterogeneous dyads. Additionally, tuned interactions of either homogeneous or heterogeneous dyads should appear as most effective’ [59]. To push beyond the 'healthy' versus 'patient' dichotomy, as a first step, it considers interactions not only within neurotypical, but also mixed dyads or groups and crucially between individuals from a certain social group (e.g. individuals from a social or a so-called neural minority). That is, the dialectical misattunement hypothesis proposes moving beyond merely contrasting—*a priori* and largely arbitrarily defined—groups of individuals toward systematically studying the multi-scale dynamics of social interaction.

A focus on interactions between persons sharing a condition, such as autism, will have a dual benefit. First, tapping into interpersonal mismatch processes might result in a more precise analysis of communication efficacy and potential breakdown mechanisms, beyond an exclusively neurobiological aetiology. Second, by taking atypical social interaction seriously, in terms of both research and practice, voice is given to the most relevant part of the population, namely those with a condition themselves. As Milton [82] put it, ‘autistic people will need to be utilising their voices in, claiming ownership of the means of autistic production, and potentially celebrate the diversity of dispositions within and without the culture’.

Nevertheless, the dialectical misattunement hypothesis eventually questions *a priori* dichotomies altogether, aiming at eventually breaking free from acknowledged weaknesses of prominent nosological^1^ disease models. As van Praag questioned (2000), ‘are the diagnostic constructs we are used to working with valid and clinically relevant or, rather, pseudo-entities; artefacts of a rigidly applied nosological doctrine’. Dialectical misattunement attempts to bypass such pitfalls by examining psychopathology as a continuum of interpersonal mismatch. In the case of autism, a straightforward way to operationalize this is via studying the interpersonal difference of autistic traits, rather than merely individual traits. For example, following this approach it has been recently shown that the more similar two persons are in autistic traits, the higher is the reported friendship quality [22]. Ultimately, as we will discuss later in this article, such a research line points toward studying psychiatric conditions transdiagnostically, as interpersonal distance in a multi-dimensional feature space.

Having said that, we still maintain that considering exclusively the social dimension of psychopathology leads to an incomplete understanding and that psychophysiological mechanisms should be addressed in parallel. Indeed, the boundary of neural, behavioural and social are in the eye of the beholder, and the understanding of the physiological mechanisms supporting social dynamics is as important as the understanding of the impact of social dynamics on individuals' psychophysiological autonomy and balance. Here, dialectical misattunement resonates with the neurodiversity paradigm which, acknowledging the need to address certain aspects on an individual basis, still views psychopathology as a human variation rather than an *a priori* disorder.

In this point, having considered situations of typical and atypical attunement between persons, and also between them and the environment, we now turn to situations where interpersonal attunement fails after having been more or less typical up to a certain point in life. An extended involuntary solitary confinement may constitute such a case. Typically, isolated individuals report multiple perceptual hallucinations—frequently of a social nature [83]. Adopting a dialectical misattunement perspective, we argue that this kind of hallucination might be a way to reduce prediction error due to a discrepancy between strong bodily expectations to (socially) interact and unexpectedly unfolding reality. Indeed, ‘Heidegger [84] provides an analysis of human existence in which being-with (Mitsein) or being-with others is part of the very structure of human existence, shaping the way that we are in the world. … In effect, one doesn't come to have a social constitution by way of interacting with others; one is “hard-wired” to be other-oriented, and this is an existential characteristic that makes human existence what it is’ [85]. Even worse, such situations may turn out to be self-enhancing, as a person who experiences extended isolation can potentially gradually develop a form of ‘social atrophy’, which in turn can contribute to further isolation and so on. ‘Social atrophy’ is a metaphor used to describe a situation in which social skills deteriorate because they are not used as much as expected—like muscle atrophy is used to describe the weakening of our muscles when they remain persistently inactive. Here, we could consider various relevant examples, such as homeless persons, institutionalized or otherwise socially excluded. Such phenomena can at times even apply to whole populations, as it has been the case with the recent repeated COVID-19 lockdowns, aiming at restricting the spreading of the pandemic. Taken together, the theoretical investigation of interpersonal misattunement and how it could be potentially ameliorated and even prevented could be relevant, not only to psychiatry, but various other fields of research and societal practice and could potentially concern each and everyone of us.

The dialectical misattunement hypothesis shares certain commonalities with approaches of different fields, such as computational psychiatry and sociology, from which it departs due to its inherently multi-scale nature. For example, leaning on predictive processing and active inference shares common ground with accounts of computational psychiatry, such as the HIPPEA ([86]; for High Inflexible Precision of Prediction Errors) and the aberrant precision one [87], which attempt to redefine autism as a deficit of domain-general information processing, explaining difficulties such as relevant to theory of mind, executive dysfunction and central coherence under a common umbrella. Notably, while these accounts constitute an important development, touching also upon certain social aspects, they still view the condition from a methodologically individualistic perspective: the deficit or difference lies exclusively in the autistic individual. A relevant account to the dialectical misattunement hypothesis within the sociological field is the ‘double empathy problem’ (DEP), which insightfully questions the ontological status of autism as articulated in prominent cognitivist accounts in favour of an interactional and relational one [88]. Although the DEP has been an important idea, helping shift the perspective to autism away from methodologically individualistic approaches, it still remains relatively agnostic about the key psychophysiological processes.

In a nutshell, due to various conceptual and methodological, but also societal constraints, sociological and psychophysiological processes have been largely studied in isolation [89,90]. In fact, it has been suggested that the study of the single brain can be in-principle sufficient to understand (social) cognition [91]. The dialectical misattunement hypothesis aims at dialectically synthesizing the levels of the individual and the collective through a principled approach. Adopting a Vygotskian perspective, it considers the historical and social construction of the atypical self, while adhering to a scientific understanding of not only interpersonal but also interrelated neurobiological mechanisms. Taken together, the dialectical misattunement hypothesis hopes to serve as a tool for the theoretical, methodological and empirical study of the multi-scale dynamics of psychopathology pushing beyond both reductionistic and descriptive accounts.

## 3. Collective psychophysiology: measuring and analysing interpersonal (mis)attunement from a second-person perspective

In the previous sections, we reviewed interpersonal attunement and misattunement in social interactions from various angles. Yet, however informative conceptual work might be, an effort to truly go beyond the individual will remain incomplete until put to the test empirically, not only in the laboratory, which allows for great experimental controllability, but also where it really matters, in real life. To this end, here we describe the paradigm of collective psychophysiology, which enables the measurement and analysis of the multi-scale processes of social interaction [20,22,76,92–100]. More concretely, it, first, embraces the dialectic between the individual and the collective via embedding empirical studies within the context of social interactions, while, second, it synthesizes well-established empirical practices, ranging from multi-person observational and phenomenological approaches to multi-modal neurobehavioral recordings in order to study social phenomena across scales and contexts.

Indeed, crucial aspects of interpersonal attunement and misattunement might not be always graspable by so-called spectatorial paradigms, which primarily trigger and monitor either (third-person) inferential or (first-person) phenomenological processes. By contrast—but also complementarily—to such accounts, second-person accounts emphasize the role of the real time and reciprocal dynamics of social interactions in making sense of others: ‘These accounts—sometimes contrastively described as the ‘second-person’ approach to other minds—ask whether social cognition from an observer's point of view is really the most pervasive way of knowing other minds and suggest that social cognition may be fundamentally different when we are actively engaged with others in ongoing social interaction, i.e. when we engage in social cognition from an interactor's point of view’ ([101]; see also [76,102,103]). In fact, it has been suggested that social interaction in and of itself might even constitute—rather than merely contextualize or enable—social cognition (cf. participatory sense making; [24,104]). In our opinion, what is crucial in this debate is to preclude the empirical paradigm from *a priori* prioritizing selected aspects of human experience and psychopathology.

**Figure 1.**
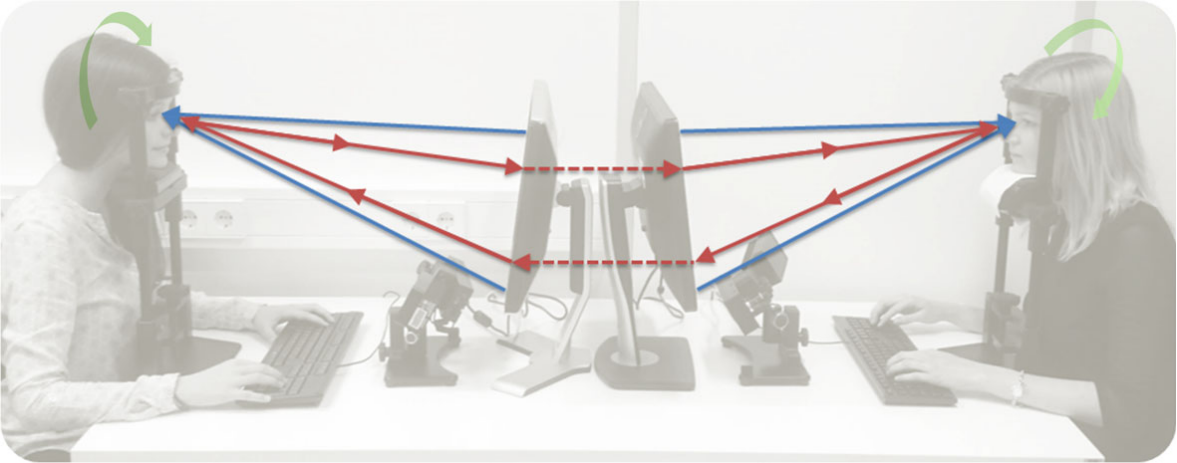
Two-person psychophysiology: this framework allows for the systematic study of the interrelation between psychophysiological and social processes in dyadic face-to-face real-time social interactions; external linear (blue) arrows, controlled environmental information; cyclic (green) arrows, intrapersonal processes; internal linear (red) arrows, social interaction loop mediated by a micro-camera communication system (adapted from Bolis & Schilbach [93]). (Online version in colour.)

In a nutshell, collective psychophysiology comprises a recent empirical paradigm that synthesizes and extends experimental and observational approaches, ranging from strictly structured and free viewing tasks to real-time social interaction and real-life aspects. This allows for the controlled recording of high-resolution datasets from multiple modalities, while retaining adequate degrees of ecological validity. In figure 1 one can find an example of a two-person psychophysiology set-up, designed for studying the multi-scale dynamics of dyadic real-time social interactions in high resolution in the laboratory.

Recording robust and meaningful datasets is the first crucial step towards capturing the essence of complex phenomena. However, the development of suitable analytical methods is of paramount importance. Typical summary measures, averaged over time, offer convenient descriptions; yet, when it comes to non-stationary processes and chaotic-like systems, such as the case of real-time social interactions, such methods fail to capture the critical aspects of the multi-scale temporal dynamics [105].

Therefore, here we anticipate experimental and computational approaches grounded in a synthesis of dynamical systems theories for formally grasping the real-time interpersonal dynamics on one hand and computational accounts of cognition for formally grasping the intrapersonal bodily processes on the other hand (for a preliminary sketch see [59,74]). Such an approach could formally show how collective dynamics in social interactions (e.g. quantified by dynamical systems trajectories) are potentially tracked and enacted by the individual (e.g. quantified by active inference states) and vice versa. Put simply, we view both ‘low-level attunement’, which plays out in relatively short spatio-temporal scales (e.g. interpersonal coupling in real-time social interaction) and ‘high-level attunement’, which is achieved through the interpersonal alignment and deepening of (inter-)bodily structures toward increasing abstraction beyond the ‘here and now’ (e.g. formation of social norms). Having said that, low- and high-level attunement should not be viewed as parts of a dichotomy, but rather within their dynamic interrelation [20,106].

Indeed, recent empirical work has demonstrated that distributed neural dynamics integrates information from ‘low-level’ sensorimotor mechanisms and ‘high-level’ social cognition to support the realistic social behaviours that play out in real time during interactive scenarios [107]. The study of such interplay between real-time ongoing social dynamics at the sensorimotor level, and slower paced representational aspects of social negotiation require new experimental paradigms. This goes from human–machine interaction [108] to human–human interaction [109], and for the latter a specific neuroimaging technique has contributed to the burgeoning field of interactive social neuroscience: hyperscanning—the recording of multiple brains simultaneously [99]. In two decades, this new approach has not only demonstrated that social perception and social interaction were leading to different neural correlates [110–112] but also uncovered new interpersonal signatures: inter-brain synchronization [9,113]. Few neurocomputational models have tried to explain social interaction dynamics, most of them focusing on sensorimotor interaction [110,111,114]. Integrating the cognitive part of social cognition thus presents a great challenge for future studies [59].

In a nutshell, our conceptual analyses focused on the importance of studying *interpersonal (mis)attunement* in social interactions, describing *second-person neuroscience* and *collective psychophysiology* as a most promising methodology to this end. In the next section, we introduce the paradigm of *inter-personalized psychiatry*, which will aim at the clinical and scientific assessment, monitoring and treatment of not only personal, but also interpersonal parameters and states, potentially resulting in a complementary redefinition of current psychiatric conditions.

## 4. Towards an *inter-*personalized psychiatry: the example of the *autism space*

The here-described approach to interpersonal attunement points toward a psychiatry that embraces the individual in all its dimensions, in particular focusing on the interpersonal aspects of mental health, which could be seen as pointing toward the development of an *inter-*personalized psychiatry. The above-mentioned dialectical misattunement hypothesis has considered psychopathology as a process that unfolds between the level of the individual and the collective and construes psychopathology at least in part as a social interaction mismatch rather than a brain disorder *per se*. Here, we extend this discussion in order to motivate further conceptual, empirical and computational directions which might allow for a principled redefinition of psychiatric *spectrum conditions* as *space conditions*, thereby doing justice to their relational, multi-dimensional and multi-scale nature. To this end, we take autism spectrum as a paradigm example and apply an extended version of what has been described as generative embedding [115].

Generative embedding is a data analysis approach that consists of two steps, namely the generative modelling step, which aims at modelling mechanisms of phenomena, and the discriminative step, which aims at capturing discriminative information in the modelled data. The generative modelling step serves as a meaningful dimensionality reduction from the measurement to a latent space (cf. predictive processing and active inference). The discriminative step deploys machine learning for group classification (e.g. autistic and non-autistic) and feature selection (i.e. selection of crucial parameters for distinguishing between groups). Additionally, this step makes it also possible to adopt an unsupervised scheme, i.e. learning directly based on unlabelled information, allowing for a data-driven identification of statistically meaningful sub-groups without relying on *a priori* categorical assumptions.

**Figure 2.**
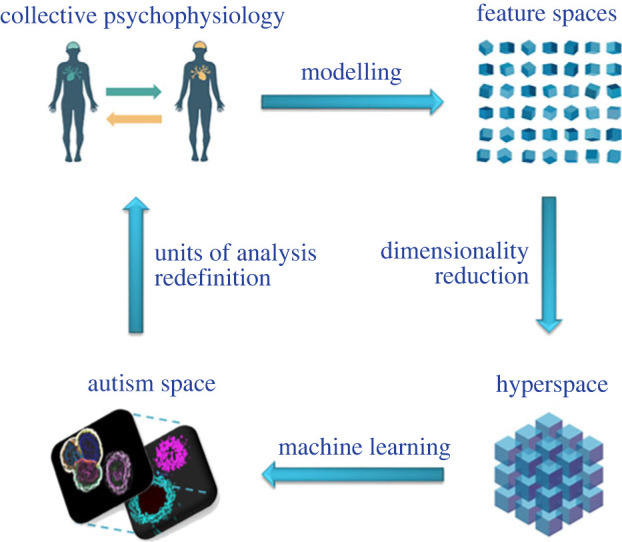
Proposed spiral pipeline (from top-left, clockwise to bottom-left): (*a*) hypotheses-specific multi-modal data is acquired within a collective psychophysiology framework; (*b*) computational modelling yields multiple parameter spaces; (*c*) hypotheses-specific spaces are merged into a single hyperspace; (*d*) dimensionality reduction yields a multi-level relational feature space (cf. autism space) including both individual and relational factors. (adapted from Bolis [22]). (Online version in colour.)

Based on generative embedding, we now delineate a research line consisting of four core steps (figure 2). In the first step, the units of analysis are explicitly defined, and subsequently collective psychophysiology data is acquired in different (social) interaction contexts, aiming at probing different mechanisms (cf. phenomenological [first-person], interactional [second-person] and inferential [third-person] phenomena). In a second step, by repeatedly applying generative modelling, the raw data is projected onto several low-dimensional parameter spaces, one for each experiment. In a third step, the separate parameter spaces are concatenated to form a single hyperspace, by virtue of bringing together all calculated parameters and states. In the fourth step, discriminative approaches are performed for identifying the crucial independent dimensions of the hyperspace, yielding a formalized definition of the feature space. By repeating this cycle of experiments and data analyses, an (increasingly informed) multi-dimensional feature space (cf. autism space within a broader inter-condition space) is constructed, being motivated by, and resulting in, increasingly sophisticated units of analyses and experimental designs. This pipeline is thought to perform repeated cycles: from the definition of the units of analysis and experimental measurements to computational modelling, machine learning, data interpretation and back to the redefinition of the units of analysis. In other words, this research line is meant to perform a periodic movement, without returning to the same point.

In short, the proposed procedure is expected to delineate a dynamic multi-scale space of conditions that will be populated by not only individual parameters and states, but also interactive and relational ones [59,98], potentially encapsulating fuzzy clusters of (sub-)conditions [116]. Our assumption, here, is that fine-grained, objective measurements of social interactions could help to identify, in a data-driven manner, how belonging to a certain diagnostic category possibly reflects certain social interaction patterns. In doing so, it may also turn out that transdiagnostic markers of social impairments are identified, a fact which might be even more important for treatment than establishing whether someone belongs to a certain diagnostic group. In fact, such an approach may not necessarily be grounded in current diagnostic criteria (allowing for classification in line with existing categorical knowledge), but on the contrary, by following an unsupervised data-driven approach, inherent biases of diagnostic manuals could potentially be ameliorated (by virtue of potentially unveiling novel transdiagnostic avenues). Here, we draw inspiration from, but also extend toward an interpersonal dimension, the vision of Stephan and Mathys [44]: ‘The hope for the future is that the delineation of patient sub-groups characterized by different disease processes, as indexed by mechanistically interpretable models, will allow for principled predictions about individual treatment and, eventually, pave the way towards a new nosology’.

Such a multi-scale account of focusing on real-time social interactions and real-life relationships will be critical, as it has been suggested that social interactions may entail processes fundamentally different than situations of passive social observation [76], while psychiatric disorders, thought of as disorders of social interaction, might be more prominent or may fully manifest in real-time social interactions and real-life relationships [20,59,101,117].

Here, it is important to emphasize the dynamicity of such a definition of a multi-dimensional feature space of conditions. Taking into account that people, their interrelationships within society, as well as the concepts of psychiatric conditions themselves are all dynamic processes, such a procedure does not aim at concluding with a fixed definition, but on the contrary at allowing for continuously capturing the essential dynamics of the co-development of biological processes, individual persons, their interactions and relationships, as well as related emerging concepts, such as social norms in their historical movement and inherent contradiction. It is interesting to note here that our here-described empirical and computational scheme could theoretically accommodate all the above-mentioned processes, as all of them, from neurobiology to social norms, can be thought of as a dynamical process, potentially modelled as Bayesian states. Consequently, this kind of analysis will include not only a given patient, but also her social interactions with others, ranging from significant others to the therapists themselves. In a nutshell, a combined approach of dynamical systems analyses, computational modelling and machine learning will help to provide a mechanistic account of interpersonal misattunement, that is, when, how and why social interactions go wrong, across scales and levels of description, by virtue of bridging the gap between social interaction, behaviour and biology.

Our formal approach eventually aims at embracing the individual not only personally but also interpersonally, pointing toward the development of an *inter-*personalized psychiatry. In fact, we deliberately introduce the new term of inter-personalized psychiatry—in contradistinction to *personalized psychiatry*—to make a case for taking social interactions seriously across all domains of research and practice, from conceptual, empirical and computational analyses, to clinical and societal practice. The promising notion of personalized medicine has been built around the premise that ‘an individual's unique physiologic characteristics play a significant role in both disease vulnerability and in response to specific therapies’ [118]. While the idea can be traced back to, at least, Hippocrates, according to whom ‘it is more important to know what sort of person has a disease than to know what sort of disease a person has’ (quoted in [119], the term has gained a revitalized attention recently due to developments of sophisticated methodologies of acquisition and analysis of big (biological) data, e.g. genomics, proteomics and metabolomics.

Here, our approach of inter-personalized medicine goes directly beyond the individual, by formally embracing the unique interactional and relational characteristics of dyads and groups of persons, while pushing toward the formal development of ‘sociomics’. Here, we view sociomics as the discipline which will address the blind spot of the systematic acquisition and analysis of real-time social interaction and real-world social relationship data. This, we foresee, will lead to a revolutionization of established methodologies targeting the individual (cf. biomarkers, biofeedback, biometrics, self-report questionnaires and brain stimulation) via widening the spotlight to include the dyad and the social group (cf. sociomarkers, cross-brain neurofeedback or sociofeedback, sociometrics, dyad-report questionnaires and multi-brain stimulation [22,59,113,120–123]), akin to the development of hyperscanning as an extension to single-brain imaging [99].

Taken together, developing the paradigm of inter-personalized psychiatry will help bring the measurement of social interaction to the foreground, allowing for the quantitative assessment of psychiatric conditions both within and across interacting individuals. This would offer an additional level of description that has a long-standing history in psychiatry (assessment of so-called psychomotor symptoms), but which can benefit from novel technologies and the integration of interactional and relational data [59,98,117,124]. In other words, this approach could potentially offer a suite of socio- and biomarkers for psychiatric conditions and could help to stratify patients and interaction profiles.

Here, we should note that, critically, in addition to certain diagnostic criteria being met that indicate the presence of a disease entity, in psychiatric practice, the subjective suffering reported by individuals (or those in close contact with them) is also assessed. Importantly, it is also assessed whether social participation is reduced as a consequence of the occurrence of a psychiatric condition. Consequently, here we are not suggesting eliminating these established ways of diagnosing and treating in psychiatry. What we are suggesting is that finding ways to quantitatively assess social interactions, without neglecting qualitative dimensions, could help to elucidate the interrelating social, behavioural, psychological and neurobiological mechanisms. Such a development, we argue, will be an important contribution by virtue of generating inter-personalized prediction models for not only advancing our scientific understanding, but also assisting clinical practice and supporting real-life processes, when needed.

Of note, while our inter-personalized approach to psychiatry is in agreement with the current dominant view that mental disorders entail neurobiological, psychological, as well as social dimensions, it is in sharp contrast with its bottom-up hierarchical assumption about the interrelation of those dimensions. As Priebe and colleagues insightfully state [125] : ‘[the prevailing paradigm] regards neurobiological aspects as the basis of disorders, which are then expressed in psychological symptoms influenced and managed within a social context. Neurobiological findings tend to be taken as explanations for disorders. Neurobiological processes have been proposed as explanations for how and why interventions work, including psychotherapy.’ Here, our aim is to turn orthodox medicine on its head, by suggesting that what we need to do is to systematically look at the interpersonal fit of persons and how they establish fulfilling social connections, without neglecting the relevant neurobiological processes, or in other words to naturalize the development of the human mind and psychopathology, without neglecting their cardinal social origins. In a nutshell, by bringing together and formally integrating the studies of the individual and the social, inter-personalized psychiatry aspires to go beyond both of them.

## 5. Interpersonal (mis-)attunement in society

Taken together, our approach to interpersonal attunement in and through social interaction aims at providing the conceptual and methodological tools in order to delineate the multi-scale dynamics of the dialectic between social interaction and the mind. For instance, developmental studies of interpersonal attunement, quantified by collective psychophysiology, interpersonal predictive processing and active inference, as well as dynamical systems as discussed above, might provide novel insights about how interpersonal dialogue is progressively transformed into internal speech. This sort of development could eventually lead to a mechanistic account of abstract concept formation across development, potentially unveiling the social and embodied origins of the human self, while also facilitating the development of artificial intelligence (cf. symbol emergence; [126]).

Perhaps, most importantly, the implications of an ecologically valid, real-world approach to social interaction reach further than any particular field of research. As Vygotsky stated, ‘we cannot master the truth about personality and personality itself as long as mankind has not mastered the truth about society and society itself’ [127, p. 342]. Taking the collective dimension of human becoming in its interrelation with the individual seriously, as a dialectic between inter- and intrapersonal attunement (without neglecting the constructive tensions, conflicts and struggles of misattunement; [20]), points toward concrete directions for societal practice.

For instance, with regard to pedagogy, our approach speaks to an interactive, collaborative and participant-oriented learning framework as opposed to a commonly deployed hierarchical and competitive one [20]. It is also in line with a legal system which takes into account not only individual but also collective responsibility, while rejecting certain rehabilitation practices, such as solitary confinement, as literally dehumanizing. With regard to clinical practice, we suggest an inter-personalized psychiatry, which will be systematically monitoring, evaluating and modulating not only intrapersonal (e.g. psychophysiological and phenomenological), but also interpersonal processes across various contexts. This could include social interactions and interpersonal relationships with the therapist, significant others, within the family, school or work, but also the broad link to society, as this can help align expectations and as such may improve understanding of others.

Here, we wish to emphasize the relation between psychotherapist and patient, whose interpersonal (mis)match may require closer evaluation, as not every psychotherapist might be effective for every patient [20]. Along these lines, psychotherapy in a dyadic setting could be investigated by means of interaction-based phenotyping and psychophysiology [59,98,124], which could help to identify communication problems between patient and therapist in order to analyse them in terms of underlying mechanisms, so that the patient (but also the therapist) can learn from them. Furthermore, in psychotherapy in a group setting, a formal communication model could be used in order to explain how persons (of different conditions) perceive the world and others. This could help to explain when and how communication breaks down and what can be done to prevent this. Additionally, our approach points from current individualistically oriented treatment options, such as biofeedback, toward interpersonal ones, such as sociofeedback—from learning to regulate intrapersonal functioning to learning to regulate interpersonal functioning [59] and the use of social niches to stabilize mental health [128].

Before concluding, we would like to underline the interrelation between psychological and socioeconomic processes in social interactions, such as the generation and perpetuation of social stigma and inequality. Social stigma may not function only as a cause of inequality, but also as a result thereof. Therefore, a pragmatic approach to mental health should aim at balancing structural asymmetries within actual society. This could include, but is not limited to, reducing social exclusion, as well as facilitating housing, employment and relational seeking. Taken together, a mutually informed understanding of dysfunction in social interactions—across scales and contexts—and its biological correlates may be exactly the key to finding new, pragmatically efficient research and treatment strategy [59,101], but also reminds us of the necessity to collectively work for the societal change needed to promote well-being and mental health for all.

## Data Availability

This article has no additional data.

## Footnotes

1 Here, nosology can be defined as the branch of medical science dealing with the classification of diseases.
